# Development and Validation of the CVP Score: A Cross-Sectional Study in Greece

**DOI:** 10.3390/healthcare11111543

**Published:** 2023-05-25

**Authors:** Konstantinos Giakoumidakis, Athina Patelarou, Anastasia A. Chatziefstratiou, Michail Zografakis-Sfakianakis, Nikolaos V. Fotos, Evridiki Patelarou

**Affiliations:** 1 Department of Nursing, School of Health Sciences, Hellenic Mediterranean University, 71410 Heraklion, Greece; apatelarou@hmu.gr (A.P.); mzografakis@hmu.gr (M.Z.-S.); epatelarou@hmu.gr (E.P.); 2 Cardiac Surgery Intensive Care Unit, Agia Sophia, General Pediatric Hospital of Athens, 11527 Athens, Greece; a.chatziefstratiou@yahoo.gr; 3 Department of Nursing, School of Health Sciences, National & Kapodistrian University of Athens, 15771 Athens, Greece; nikfotos@nurs.uoa.gr

**Keywords:** central venous pressure, intensive care units, questionnaire, reliability, validity

## Abstract

Although central venous pressure (CVP) is among the most frequent estimated hemodynamic parameters in the critically ill setting, extremely little is known on how intensive care unit (ICU) nurses use this index in their decision-making process. The purpose of the study was to develop a new questionnaire for accessing how ICU nurses use CVP measurements to address patients’ hemodynamics investigating its validity and reliability. A cross-sectional study was conducted among 120 ICU nurses from four ICUs of Greece. Based on a comprehensive literature review and the evaluation by a panel of five experts, a new questionnaire, named “CVP Score”, was created, having eight items. The construct validity and the reliability of the questionnaire were examined. Half of the study participants (51.7%) worked at a specialized ICU, and they had a mean [±Standard Deviation (SD)] ICU experience of 13(±7.1) years. The estimated construct validity of the newly developed tool was acceptable, while the internal consistency reliability as measured by Cronbach alpha was excellent (0.901). CVP Score had acceptable test–retest reliability (r = 0.996, *p* < 0.001) and split-half reliability (0.855). The CVP score is a valid and reliable instrument for measuring how critical care nurses use CVP measurements in their decision-making process.

## 1. Introduction

Central venous pressure (CVP) is a hemodynamic parameter that has widely been monitored in the intensive care units (ICU) for assessing cardiac function, the right atrium preload, the volume status, the fluid responsiveness in critically ill patients, and it is defined as the estimated pressure in the vena cava, so can be considered as a measurement of cardiac preload and right atrial pressure [1,2,3]. The normal range of CVP, that is measured in a supine position via a central venous catheter placed into the subclavian or internal jugular vein, is inconsistent with different depicted values, such as 2–8 mmHg [4], 0–10 mmHg [5], or 5–15 mmHg [6], available in the currently available published research. A normal CVP waveform consists of three peak (a, c, and v waves) and two descent (x and y) phases that represent pressure changes in the right atrium. The a, c, and v waves represent atrial contraction, isovolumic ventricular contraction, and atrial systolic filling, respectively. Additionally, x descent highlights the relaxation of the atrium, while y descent highlights the early ventricular filling [5].

CVP is considered as a static indicator of cardiac preload and blood returning to the right side of the heart, despite other parameters, such as systolic pressure variation, pressure pulse variation, stroke volume variation, tidal volume challenge, respiratory change in aortic blood flow, aortic blood flow peak velocity variation, respiratory changes in pre-ejection period, variation of plethysmography, and superior–inferior vena cava collapse index, that have been recognized as dynamic indexes of fluid responsiveness [7]. Even the variability in CVP values cannot reliably predict intravenous fluid therapy responsiveness among the ICU patients’ setting and the assessment of CVP values’ changes remains problematic and cannot be used to determine if a patient is fluid overloaded or dehydrated [7]. At the same time, the existing evidence strongly suggests the inadequacy of single CVP measurements to guide fluid administration and resuscitation clinical decisions. It seems that only extreme measurements of CVP either low or high could guide fluid administration interventions in a more effective way [1].

Indeed, over the course of time, the usefulness and the accuracy of CVP as a strong hemodynamic and endovascular volume index has been debated and the currently available published research reveals no absolute correlation between CVP values and the total blood volume present in human circulation [8]. Osman et al. [9], in their retrospective study of 96 patients conducted in a single 24-bed medical ICU, reported that a CVP value less than 8 mmHg has a positive and negative predicted value of 51% and 65%, respectively. Additionally, Marik et al. [10] by using meta-analysis of 24 studies with a sample of 803 critically ill patient concluded that CVP is unable to predict fluid responsiveness and there is no adequate proof to support the practice of using these parameters as a guiding index of fluid administration for therapeutic purposes. In line with the above-mentioned findings, a recent single-center study of 97 critically ill patients who underwent allogeneic renal transplantation showed the superiority of stroke volume variation in guiding fluid management compared with CVP, reporting that fluid management guided by stroke volume variation monitoring, compared with CVP values evaluation, can be associated with optimal outcomes, such as the reduction in intraoperative fluid volume, the improvement of the kidney perfusion, and the promotion of postoperative recovery [11]. In favor of these data, the European Society of Intensive Care Medicine does not recommend the CVP as a measure to guide the fluids’ administration and to predict the patient responsiveness to fluid therapy [8]. It seems that many inherent weaknesses of the CVP affect negatively its reliability, given that CVP values are altered by many of parameters outside the circulating blood volume, such as venous compliance, systematic vascular resistances, pulmonary hypertension, tricuspid valve insufficiency, heart failure, cardiac dysrhythmias, and conditions associated with increased intrathoracic pressure, including cardiac tamponade, tension pneumothorax, positive pressure mechanical ventilation, and positive end-expiratory pressure [12]. Additionally, CVP can be altered due to the patient body posture [13] or the presence of a valve disease [7].

Despite the inherent limitations of the CVP, that generate its sensitivity in a variety of parameters and clinical disorders, and the presence of other methods for volume status estimation and guidance to fluid administration in the clinical setting, such as trans-esophageal echocardiography and ultrasound-guide techniques, CVP remains the most frequent estimated hemodynamic parameter in the critically ill setting by intensivists and critical care nurses [14]. In Greece, recently the CVP measurement and estimation are among the legal professional duties of the critical care nurses [15]. It is possible that the minimal and low-cost apparatus, the easiness to be measured, and its satisfactory predictive value concerning extremely low of high values [1] are the main factors that could explain, despite the current research trends and data, its classification among the most frequently hemodynamics parameters that measured among critically ill patients in order to guide clinical decisions regarding the fluids’ response and therapy.

Although many studies have been carried out in order to determine the accuracy and the reliability of CVP value for the estimation of critically ill volume and hemodynamic status [9,10,16], nothing we know regarding the extent in which critical care nurses, use CVP measurements in their decision-making process for the optimal volume, hemodynamic, and cardiovascular management of the critically ill patients, given that CVP measurements are both a frequent intervention and a legally recognized critical care nurses’ professional responsibility. Attempting to add new research data to this body of knowledge was the aim of the present study in order to instigate the development of a new questionnaire for accessing how critical care nurses use CVP measurements to address patients’ hemodynamics and volume status. Additionally, this study aimed to investigate the validity and reliability of this newly developed questionnaire.

## 2. Materials and Methods

### 2.1. Study Design and Participants

A cross-sectional, validation study was conducted among critical care nurses from four ICUs of two general tertiary hospitals of Greece. Being a critical care nurse was the inclusion criterion of the study. On the other hand, nurses who were unwilling to give their written consent to participate in our study and those with uncompleted filled out questionnaires were excluded. Based on these exclusion criteria, the final study sample was 120 critical care nurses. Data collection took place during a three-month period (from August to October 2018). This sample size meets the minimum requirement for the instrument validation process for at least 10 participants per questionnaire item [17].

### 2.2. Content Validity

Aiming to create the new questionnaire, named “CVP Score”, a comprehensive literature review was conducted. In the currently available published research, we did not find any instrument which measure how nurses use CVP values in order to manage the critically ill patients and to determine their nursing care plans. At the first phase of the development of the CVP Score, 10 items have been selected for the entire questionnaire. Each item was a full sentence of specific interventions related to how nurses use CVP values in order to determine their care planning and to make clinical decisions for the critically ill patient management. Each item of the questionnaire could be answered using a 4-point Likert scale from “Never” (1 point) to “Always” (4 points).

Assessing the content validity of the new-developed questionnaire, this tool was evaluated by a five-expert panel, consisting of 2 ICU nurses, 1 intensivist, and 2 researchers with significant scientific work on intensive and critical care nursing. Each item of the questionnaire was graded by the experts as “essential”, “useful but inadequate”, or “unnecessary”. All experts’ evaluations were taken into account and finally 2 items were excluded from the questionnaire, given that their content validity ratio was lower than 0.99, according to the Lawshe Table for Minimum Values of content validity ratio [18,19]. Specifically, the content validity ratio of the first and second excluded items were 0.66 and −0.2, respectively. Subsequently, 10 people from the general population and out of our study sample provide feedback on the 8-item questionnaire, evaluating the linguistic clarity of the tool.

As presented in Tables S1 and S2, the final 8-item tool included the following questions. (1) I routinely measure CVP, two or more times during my shift; (2) I measure central venous pressure in each case of patient hemodynamic instability; (3) I estimate the fluid volume excess or deficit based on CVP values, more than the others hemodynamic parameters; (4) I plan fluid administration in low CVP values, independently of the patient blood pressure and heart rate (beats/min); (5) I plan to give diuretics and/or to limit fluid administration, independently of the patient blood pressure and heart rate (beats/min); (6) I plan my interventions (fluids administration—limitation, diuretic administration) taking into account the isolated CVP values, more than their trends—changes; (7) the inability for CVP measurement (e.g., absence of a central venous catheter, blocked lumens) negatively affects me to estimate patient hemodynamics; and (8) I predict patient fluid responsiveness by CVP values.

### 2.3. Data Collection

Structured face to face interviews were conducted for data collection purposes, among 120 critical care nurses from two ICUs of two general tertiary hospitals of Greece, using the “CVP Score” and a second short questionnaire on basic participants’ demographics. The CVP score ranged from 8 to 32. The high values of the CVP Score indicate high use of CVP for the nursing assessment of patients’ hemodynamic and volume status, while low values are indicative that nurses considered CVP as a poor clinical tool in order to estimate patients’ hemodynamics and to plan their provided care based on its values. An optimal cut-off point could be the median value of CVP Score and in our previous manuscript [20] we had defined the value of 16 as the value that marks the high (>16) and low (≤16) values of the CVP Score. At the second stage (second assessment), the participants re-answer the questionnaire through phone interviews, after one month from the first assessment, using the same order, to avoid memory effect on test and retest measurements.

Furthermore, data gathering purposes were served through a short questionnaire on basic participants’ demographic characteristics, such as age, gender, educational level (undergraduate and postgraduate), experience as clinical nurse, experience in the ICU setting and ICU type (general or specialized)

### 2.4. Ethics

Written permission was given from the ethics committee of both of the hospitals (234/18-07-2018 and 36382/31-07-2018). Precautions took place to protect the privacy and anonymity of the participant subjects and the confidentially of their data and information, while participants gave their sign informed consent. The collected data were used only for the purpose of the present study. All the stages of the research were carried out in full accordance with the ethical standards of the Helsinki Declaration of 1975, as revised in 2013.

### 2.5. Statistical Analysis

Quantitative and qualitative variables were expressed as mean [±Standard Deviation (SD)] values and absolute–relative frequencies, respectively. Construct validity was described by calculating the Pearson’s correlation coefficient r of the scores of the participants’ responses to an item with their total scores. The Cronbach alpha coefficient was calculated for the internal consistency reliability of the entire questionnaire. Pearson’s rank correlation coefficient was performed to measure the level of agreement between responses at test and re-test, while the Spearman–Brown formula was used for computing the split-half reliability. The IBM SPSS 24.0 for Window software (Armonk, NY, USA: IBM Corp) was used for our statistical analysis purposes.

## 3. Results

### 3.1. Demographics and Descriptive Statistics

As shown in Table 1, the mean (±SD) age of our study participants was 42.3 ± 6.1 years, while the majority of our sample was female subjects (71.7%), graduates of technological tertiary education (88.3%), and had no postgraduate education (66.7%). In addition, half of the study participants’ (51.7%) worked at a specialized ICU, and their mean (±SD) clinical and ICU experience was 17.3 (±6.8) and 13(±7.1) years, respectively (Table 1). Finally, the mean (±SD) and the median (Interquartile range) CVP Score were 15.8 (±5.7) and 16 (10.7), respectively.

### 3.2. Association between Variables

In a previous manuscript, we have shown the correlation between independent variables, such as gender, educational level (basic and postgraduate), ICU type, clinical experience, and experience in an ICU setting, and only the male gender was found as a significant predictor of increased CVP Score values that indicate the higher use of CVP by critical care nurses [20].

### 3.3. Construct Validity

As aforementioned and summarized in Table 2, construct validity was evaluated with the Pearson’s correlation coefficient r of the scores of participants’ responses to an item with their total scores. All the calculated values were statistically significant and each obtained Person Correlation coefficient value was greater than the critical value from the Pearson’s Correlation Table at 118 (N-2) degrees of freedom.

Furthermore, a factor analysis was performed. Specifically, the KMO measure of sampling adequacy was 0.806 and Bartlett’s test of sphericity was 648.380, df = 28, *p* < 0.001. Factor analysis indicated that there are two principal factors in the model, and these accounted for 72.24%, as presented in Table 3. The first factor (F1) includes item 1 (I routinely measure CVP, two or more times during my shift) and its contribution was 59.326%. The second factor (F2) consists of item 2 (I measure central venous pressure in each case of patient hemodynamic instability) and the variance explained by this factor was 12.920% (Table 3). Cronbach’s alpha was 0.702 and 0.251 for F1 and F2, respectively.

### 3.4. Instrument Internal Consistency Reliability

The Cronbach alpha was 0.901 for the entire questionnaire.

### 3.5. Test–Retest Reliability

**Figure 1:**
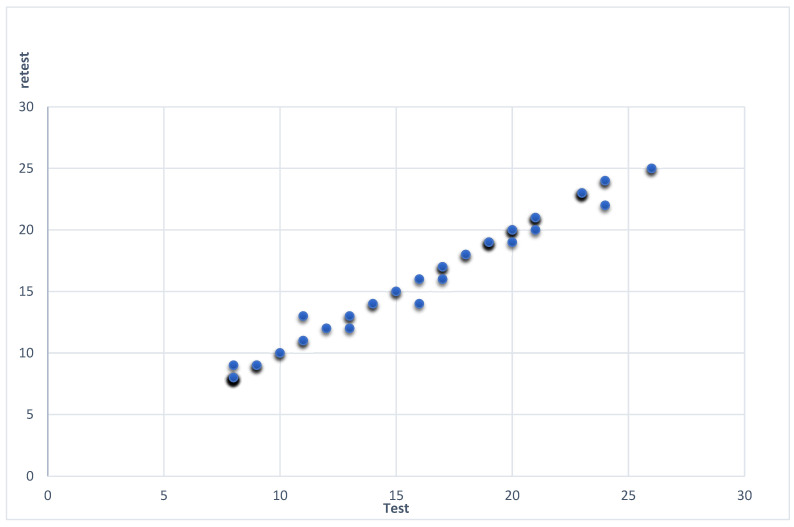
Scatter plot of the CVP Score test–retest measurements.

By using test–retest reliability coefficient correlation analysis, a high positive correlation was observed between the total scores of the two applications (r = 0.996, *p* < 0.001). The measurements of the CVP Score are depicted via a scatter plot in Figure 1. More analytically, 100 participants had the same score on both assessments (test and retest) and 20 gave different scores. From those who had different scores and answers the majority (14 participants) had higher, by one point, CVP Score between the two assessments. The remaining six critical care nurses had a two-point difference between the two measurements.

### 3.6. Split-Half Reliability

The split-half reliability was computed to be 0.855 using the Spearman–Brown formula.

## 4. Discussion

According to the main aim of the present study, we developed a new instrument in order to measure how ICU nurses use CVP in order to guide their planning, clinical decisions, interventions, and fluid administration titration for the optimal management of the critically ill patient. To the best of our knowledge, this new-developed tool named “CVP Score” was the first instrument for the above-mentioned purpose, and this tool is unique in the current literature. Another important aim of the present study was to test the validity and reliability of this newly developed instrument. According to the findings of our study, “CVP Score” is a valid and reliable instrument that intends to provide researchers a significant and acceptable tool for assessing the extent of CVP values use by critical care clinicians for ICU patients in the planning of care and decision-making.

The knowledge on how critical care nurses use CVP measurements for their care planning is of utmost importance because there is not much information on this subject. Although, it is evident that CVP is the most frequent hemodynamic parameters that are estimated in the ICU environment; at the same time, we have a full absence of unambiguous proof regarding the way of CVP values incorporation in the daily nursing clinical practice. Theoretically, a significant probability is that critical care nurses estimate CVP values but they do not use these measurements to guide their clinical decisions, based on the current available literature results. Then, again, another probability is that CVP measurements operate, in critical care nurses’ considerations and thoughts, as predictors of patients’ hemodynamics and guide for their fluid therapy. The existence of a questionnaire, such as the “CVP Score”, could provide a tool for both replying to the above questions and addressing the vision of critical care nurse on the value of CVP.

At the same time, future research projects findings with the use of the CVP score could be used productively. The mapping of the critical care nurses’ considerations of CVP values during their clinical decision-making is the first step for their educational needs’ evaluation in the context of continuing professional education. For instance, the finding that critical care nurses base, to a considerable degree, their clinical decisions on the estimated CVP values to manage the hemodynamic and volume status of ICU patients could work as an alarming sign and alongside a motivation to establish educational interventions on evidence-based nursing in the clinical setting aiming to provide documented, improved, and updated research evidence on this topic. Likewise, the research community could investigate whether CVP measurements are used and interpreted with different point of views in different ICU settings, such as general ICU or specialized ICU (cardiac surgery, neurosurgical ICU). Based on our professional experience, we can state the tendency of critical care nurses to overestimate CVP values in specialized cardiovascular ICUs, such as cardiac and cardiac surgery ICUs, but, that being said, nurses who provide intensive nursing care to patients in general ICUs often underestimate the value of CVP measurements, considering CVP as not a helpful parameter that cannot be taking into account in their clinical decision-making process regarding the prediction of the hemodynamic and overall patients’ fluid balance.

Validity is defined as the extent to which an instrument measures exactly what it is supposed to measure without mistaking it with another issue, while reliability is the extent to which an instrument gives consistent results in repeated measurements under similar conditions [17]. Assessing the validity of the “CVP Score”, it followed that there needed to be the appropriate methodology to ensure its content validity during the development of our instrument. Additionally, the evaluated construct validity of the “CVP Score”, through the correlation of each item score with the total score, emerged as acceptable [18]. Likewise, construct validity assessed by factor analysis, based on our findings of KMO and Bartlett’s tests that indicated that our sample was excellently adequate and the correlation between the data was sufficient for factor analysis, respectively [18]. The validity of our 8-item instrument was found to be 72.24%, demonstrating that CVP Score can achieve the purpose it wants to measure [18]. The main contributive factor was the item I (I routinely measure CVP, two or more times during my shift) explained the 59.326% of the total variance, while the second item (I measure central venous pressure in each case of patient hemodynamic instability) explained the remaining 12.920%.

On the other hand, assessing the reliability of our new-developed questionnaire we observed that “CVP Score” had an excellent Cronbach alpha coefficient, given that this parameter should have values higher than 0.59 and lower than 0.95 [18]. In addition, test–retest reliability was computed as acceptable and, finally, aiming to estimate the split-half reliability of our tool we found a strong positive correlation, which highlights our questionnaire acceptability [18].

Despite the significance of the present study, the main limitation was the full absence of a valid and reliable instrument which investigates the same issue. Thereafter, the examination of parameters that evaluate how accurately the tool measures the outcome it was designed to measure, the association of the tool with accepted standards, and its ability to predict future test results, such as the criterion, concurrent, and predictive validity of the “CVP Score”, respectively, were inapplicable [18,21].

## 5. Conclusions

All things considered, and according to the validity and reliability analysis of our new-developed questionnaire, this tool is a valid and reliable instrument that could be used in the critical care setting, aiming to measure the extent to which critical care nurses use CVP measurements in their clinical decision-making process for the optimal volume, hemodynamic, cardiovascular monitoring, and management of the critically ill patients. It seems that ICU clinicians and researchers could use “CVP Score” to add new data to the above-mentioned limited body of knowledge. Our study significance could be underlying by the originality of the evaluated tool, taking into account that it is the first one to serve the above-mentioned research purpose. Although, based on the current literature, the value of the CVP measurement as a reliable index of cardiovascular and intravascular blood volume status, which can guide the fluid administration therapy and predict fluid responsiveness is controversial, the measurement of CVP remains a standard professional skill and responsibility of ICU clinicians, including critical care nurses. Our study limitations show that further research is needed, using the “CVP Score” on greater samples and in different ICU settings, countries, and healthcare systems.

## Figures and Tables

**Table 1:** Demographic characteristics of study participants.

	Mean (±SD)
**Age (years)**	42.3 (6.1)
**Clinical experience (years)**	17.3 (6.8)
**ICU experience (years)**	13 (7.1)
	**n (%)**
**Gender**	
Males	34 (28.3)
Females	86 (71.7)
**Basic educational level**	
University tertiary	14 (11.7)
Technological tertiary	106 (88.3)
**Postgraduate education**	
Yes	40 (33.3)
No	80 (66.7)
**ICU type**	
General	58 (48.3)
Specialized	62 (51.7)

ICU: Intensive care unit, SD: Standard deviation.

**Table 2:** Correlation of each item score with their total scores.

		Q1	Q2	Q3	Q4	Q5	Q6	Q7	Q8	Total
Q1	Pearson Correlation	1	0.745	0.522	0.326	0.461	0.420	0.396	0.422	0.731
	*p*-value		<0.001	<0.001	<0.001	<0.001	<0.001	<0.001	<0.001	<0.001
Q2	Pearson Correlation	0.745	1	0.627	0.413	0.509	0.358	0.489	0.536	0.791
	*p*-value	<0.001		<0.001	<0.001	<0.001	<0.001	<0.001	<0.001	<0.001
Q3	Pearson Correlation	0.522	0.627	1	0.772	0.657	0.585	0.477	0.571	0.841
	*p*-value	<0.001	<0.001		<0.001	<0.001	<0.001	<0.001	<0.001	<0.001
Q4	Pearson Correlation	0.326	0.413	0.772	1	0.732	0.652	0.326	0.578	0.757
	*p*-value	<0.001	<0.001	<0.001		<0.001	<0.001	<0.001	<0.001	<0.001
Q5	Pearson Correlation	0.461	0.509	0.657	0.732	1	0.627	0.267	0.509	0.760
	*p*-value	<0.001	<0.001	<0.001	<0.001		<0.001	0.003	<0.001	<0.001
Q6	Pearson Correlation	0.420	0.358	0.585	0.652	0.627	1	0.562	0.680	0.771
	*p*-value	<0.001	<0.001	<0.001	<0.001	<0.001		<0.001	<0.001	<0.001
Q7	Pearson Correlation	0.396	0.489	0.477	0.326	0.267	0.562	1	0.668	0.681
	*p*-value	<0.001	<0.001	<0.001	<0.001	0.003	<0.001		<0.001	<0.001
Q8	Pearson Correlation	0.422	0.536	0.571	0.578	0.509	0.680	0.668	1	0.803
	*p*-value	<0.001	<0.001	<0.001	<0.001	<0.001	<0.001	<0.001		<0.001
Total	Pearson Correlation	0.731	0.791	0.841	0.757	0.760	0.771	0.681	0.803	1
	*p*-value	<0.001	<0.001	<0.001	<0.001	<0.001	<0.001	<0.001	<0.001	

**Table 3:** Exploratory factors and explained variance after rotation for the CVP score.

Total Variance Explained							
Item	Initial Eigenvalues			Extraction Sums of Squared Loadings			Rotation Sums of Squared Loadings ^a^
	Total	% of Variance	Cumulative %	Total	% of Variance	Cumulative %	Total
Q1	4.746	59.326	59.326	4.746	59.326	59.326	4.191
Q2	1.034	12.920	72.246	1.034	12.920	72.246	3.624
Q3	0.904	11.306	83.552				
Q4	0.436	5.453	89.005				
Q5	0.320	4.004	93.010				
Q6	0.256	3.194	96.203				
Q7	0.158	1.977	98.181				
Q8	0.146	1.819	100.000				

Extraction Method: Principal Component Analysis. ^a^ When components are correlated, sums of squared loadings cannot be added to obtain a total variance.

## Data Availability

The data presented in this study are available on request from the corresponding author.

## Supplementary Materials

The following supporting information can be downloaded at: https://www.mdpi.com/article/10.3390/healthcare11111543/s1, Table S1: CVP Score (in Greek); Table S2: CVP Score (in English).
