# Supplementary material for: Development and Validation of the CVP Score: A Cross-Sectional Study in Greece
Source: Healthcare (Basel). 2023 May 25;11(11):1543. doi: 10.3390/healthcare11111543 (PMC10252753; doi:10.3390/healthcare11111543)
Supplement: Supplementary file 1 [file healthcare-11-01543-s001.zip › healthcare-2364129-supplementary.pdf]

Table S1: CVP Score (in Greek)

Παρακαλώ διαβάστε προσεκτικά τις ακόλουθες προτάσεις και επιλέξτε μία από τις τέσσερις πιθανές απαντήσεις, βάσει των αποφάσεων που λαμβάνετε και των παρεμβάσεων που επιτελείτε κατά την άσκηση του κλινικού σας έργου, σχετικά με τη μέτρηση και αξιοποίηση της κεντρικής φλεβικής πίεσης

	Καθόλου	Λίγες φορές	Αρκετές φορές	Πάντα
1. Μετράω την ΚΦΠ, ως διαδικασία ρουτίνας, δύο ή περισσότερες φορές στη βάρδια μου	1	2	3	4
2. Μετράω την ΚΦΠ σε κάθε περίπτωση αιμοδυναμικής αστάθειας του ασθενούς	1	2	3	4
3. Πραγματοποιώ εκτίμηση της περίσσειας ή του ελλείμματος όγκου υγρών του ασθενούς, βασιζόμενος στην ΚΦΠ, περισσότερο από τις υπόλοιπες αιμοδυναμικές παραμέτρους	1	2	3	4
4. Προγραμματίζω χορήγηση υγρών σε χαμηλές τιμές ΚΦΠ, ανεξάρτητα από την ΑΠ και την καρδιακή συχνότητα (σφύξεις/λεπτό) του ασθενούς	1	2	3	4
5. Προγραμματίζω περιορισμό της χορήγησης υγρών ή/και χορήγηση διουρητικών σε υψηλές τιμές ΚΦΠ, ανεξάρτητα από την ΑΠ και την καρδιακή συχνότητα (σφύξεις/λεπτό) του ασθενούς	1	2	3	4
6. Προγραμματίζω τις παρεμβάσεις μου (χορήγηση – περιορισμός υγρών, χορήγηση διουρητικών) λαμβάνοντας υπόψη περισσότερο τις μεμονωμένες τιμές ΚΦΠ, συγκριτικά με τις μεταβολές της	1	2	3	4
7. Η αδυναμία μέτρησης της ΚΦΠ (π.χ. απουσία κεντρικού φλεβικού καθετήρα, μη ικανοποιητική βατότητα αυλών) με εμποδίζει σημαντικά να εκτιμήσω την αιμοδυναμική κατάσταση του ασθενούς	1	2	3	4
8. Μέσω της τιμής της ΚΦΠ προβλέπω την ανταπόκριση του ασθενούς στη χορήγηση υγρών	1	2	3	4
Επεξήγηση συντμήσεων<br>ΑΠ: Αρτηριακή πίεση, ΚΦΠ: Κεντρική φλεβική πίεση				

Table S2: CVP Score (in English)

Please, read carefully the following sentences and choice the most appropriate answer, based on your clinical decision-making process and the interventions which you carry out during your professional work, regarding the measurement and use of the central venous pressure.

	Never	Sometimes	Usually	Always
1. I routinely measure CVP, two or more times during my shift	1	2	3	4
2. I measure central venous pressure in each case of patient hemodynamic instability	1	2	3	4
3. I estimate the fluid volume excess or deficit based on CVP values, more than the others hemodynamic parameters	1	2	3	4
4. I plan fluid administration in low CVP values, independently of the patient blood pressure and heart rate (beats/min).	1	2	3	4
5. I plan to give diuretics and/or to limit fluid administration, independently of the patient blood pressure and heart rate (beats/min).	1	2	3	4
6. I plan my interventions (fluids administration – limitation, diuretic administration) taking into account the isolated CVP values, more than their trends - changes	1	2	3	4
7. The inability for CVP measurement (eg absence of a central venous catheter, blocked lumens) negatively affects me to estimate patient hemodynamics	1	2	3	4
8. I predict patient fluid responsiveness by CVP values	1	2	3	4
CVP: Central venous pressure				
